# *Plasmodium falciparum msp1*, *msp2 *and *glurp *allele frequency and diversity in sub-Saharan Africa

**DOI:** 10.1186/1475-2875-10-79

**Published:** 2011-04-06

**Authors:** Felista Mwingira, Gamba Nkwengulila, Sonja Schoepflin, Deborah Sumari, Hans-Peter Beck, Georges Snounou, Ingrid Felger, Piero Olliaro, Kefas Mugittu

**Affiliations:** 1 Dares Salaam University College of Education P.O.BOX 2329, Dar es Salaam, Tanzania; 2 Department of Zoology and Wildlife Conservation, University of Dar es Salaam, PO Box 35064, Dar es Salaam, Tanzania; 3 Swiss Tropical and Public Health Institute, Socinstrasse 57, CH 4002, Basel, Switzerland; 4 University of Basel, Petersplatz 1, 4003 Basel, Switzerland; 5 Ifakara Health Institute, P. O. Box 74 Bagamoyo, Tanzania; 6 UPMC/INSERM UMR S 945, Faculté de Médecine Pitié-Salpêtrière Université Pierre & Marie Curie, 91 Boulevard de l'Hôpital, 75013 Paris, France; 7 UNICEF/UNDP/World Bank/WHO Special Programme for Research and Training in Tropical Diseases, World Health Organization, Room CA 1118, Centre Casai, 51-53 Avenue Louis Casai1216 Cointrin, Geneva, Switzerland; 8 Centre for Tropical Medicine and Vaccinology, Nuffield Department of Medicine, University of Oxford, Churchill Hospital, Oxford OX37LJ, UK

## Abstract

**Background:**

The efficacy of anti-malarial drugs is assessed over a period of 28-63 days (depending on the drugs' residence time) following initiation of treatment in order to capture late failures. However, prolonged follow-up increases the likelihood of new infections depending on transmission intensity. Therefore, molecular genotyping of highly polymorphic regions of *Plasmodium falciparum msp1*, *msp2 *and *glurp *loci is usually carried out to distinguish recrudescence (true failures) from new infections. This tool has now been adopted as an integral part of anti-malarial efficacy studies and clinical trials. However, there are concerns over its utility and reliability because conclusions drawn from molecular typing depend on the genetic profile of the respective parasite populations, but this profile is not systematically documented in most endemic areas. This study presents the genetic diversity of *P. falciparum msp1, msp2 *and *glurp *markers in selected sub-Saharan Africa countries with varying levels of endemicity namely Malawi, Tanzania, Uganda, Burkina Faso and São Tomé.

**Methods:**

A total 780 baseline (Day 0) blood samples from children less than seven years, recruited in a randomized controlled clinical trials done between 1996 and 2000 were genotyped. DNA was extracted; allelic frequency and diversity were investigated by PCR followed by capillary electrophoresis for *msp2 *and fragment sizing by a digitalized gel imager for *msp1 *and *glurp*.

**Results and Conclusion:**

*Plasmodium falciparum msp1, msp2 *and *glurp *markers were highly polymorphic with low allele frequencies. A total of 17 *msp1 *genotypes [eight MAD20-, one RO33- and eight K1-types]; 116 *msp2 *genotypes [83 3D7 and 33 FC27- types] and 14 *glurp *genotypes were recorded. All five sites recorded very high expected heterozygosity (H_E_) values (0.68 - 0.99). H_E _was highest in *msp2 *locus (H_E _= 0.99), and lowest for *msp1 *(H_E _= 0.68) (P < 0.0001). The genetic diversity and allelic frequency recorded were independent of transmission intensity (P = 0.84, P = 0.25 respectively. A few genotypes had particularly high frequencies; however the most abundant showed only a 4% probability that a new infection would share the same genotype as the baseline infection. This is unlikely to confound the distinction of recrudescence from new infection, particularly if more than one marker is used for genotyping. Hence, this study supports the use of *msp1, msp2 *and *glurp *in malaria clinical trials in sub-Saharan Africa to discriminate new from recrudescent infections.

## Background

The efficacy of anti-malarial drugs in endemic areas is assessed over a follow-up period of 28 to 63 days, depending of the drug's residence time in the organism, following initiation of treatment. While a longer follow-up allows capturing more late failures [1] the likelihood of re-infection increases in a way that is dependent upon the intensity of transmission in the study area. Comparing molecular genotypic pattern of pre-treatment (baseline) and recurrent infections provides a means to help characterize the recurrent parasites as a recrudescence, i.e. a true failure, or a new infection (either from pre-existing liver infection or a newly established infection from an infected mosquito bite), i.e. a successful treatment.

Several *Plasmodium falciparum *genes show extensive genetic polymorphism. This phenomenon is exploited for genetic finger printing and for assessing parasite population dynamics. For instance, high polymorphism has been shown in *msp1*, *msp2 *and *glurp *genes in different geographical locations in malaria endemic areas [2-7]. The probability of a patient, particularly in areas of intense transmission, to be newly infected with a parasite possessing an identical genotype to the former infection is low [8]. Therefore, comparing the genotypes of the three loci at baseline and at the time of parasite recurrence would be expected to discriminate between recrudescent and new infections [8,9]. Numerous clinical drug trials have applied this approach to correct the outcomes of drug efficacy studies [10-17].

However, the discriminating power of different markers is dependent on the extent of allelic diversity and on the frequency of each allele within the population under study. Indeed, recurrent episodes after treatment can be reliably classified as recrudescence or re-infections if the frequencies of the *msp2*, *msp1*, and *glurp *alleles, as detected by the genotyping protocol employed, are known. It is obvious that a second infection appearing during follow-up after the first has been apparently cleared can be erroneously classified as recrudescent if some alleles predominate in the population or if heterozygosity is low, because under these circumstances a new infection would be more likely to share the same genotype as the baseline infection. This will lead to an over-estimation of treatment failures and consequently unnecessary treatment policy changes.

The allelic diversity of *msp2 *has been observed to be high in some areas, such as in the Kilombero valley in Tanzania (82 *msp2 *alleles) [2], Papua New Guinea (42 *msp2 *alleles) [18], Ghana (164 *msp2 *alleles) [19] and Côte d'Ivoire (50 *msp2 *genotypes) [20]. Conversely, there is very limited information on *msp1 *or *glurp *diversity across sub-Saharan Africa. This study presents the *msp1, msp2 *and *glurp *genetic diversity and allele frequencies in five Sub-Saharan African countries with different transmission intensities namely, Malawi, Tanzania, Uganda, Burkina Faso and São Tomé.

## Methods

### Study area and design

This study is part of two artemisinin combination therapy (ACT) trials that took place in sub-Saharan African regions with different malaria transmission intensities as detailed below;

(i) Randomized, double-blinded artemisinin-combination trials sponsored by the UNICEF/UNDP/WORLD BANK/WHO Special Programme for Research and Training in Tropical Diseases (WHO/TDR) between 1999 and 2000 in Burkina Faso, Gabon, The Gambia, São Tomé, Senegal, Uganda, Malawi and Kenya. The drugs tested in these trials were amodiaquine, sulphadoxine-pyrimethamine (SP) or chloroquine (CQ) alone or in combination with artesunate. Due to time and financial limitations only a subset of samples were analysed from only four of these countries (Malawi, Uganda, Burkina Faso and São Tomé) and used in the current study.

(ii) A trial conducted by the Ifakara Health Institute (IHI) and Swiss Tropical and Public Health Institute (Swiss TPH) in Tanzania in 1996. Drugs tested in this study were CQ alone vs. artemether-lumefantrine (AL).

The study site characteristics, methodological and clinical findings are described elsewhere [13,21,22]. For all the studies ethical approval was obtained from relevant local and external ethics committees. Prior to recruitment, informed consent was obtained from the children's parents or guardians. Blood samples from participant patients were collected on IsoCode™ Stix (in WHO/TDR trials) or as whole blood (in IHI/Swiss TPH trial) on day 0, 7, 14, 21 and 28. A total of 780 baseline pre-treatment samples (Day 0) were randomly selected for genotyping, n = 180 from each study site except Tanzania (n = 60).

#### Molecular genotyping

DNA was extracted from IsoCode™ Stix (at IHI) by boiling (for the WHO/TDR trial samples) as described by manufacturer or by the standard phenol-chloform method (at Swiss TPH) from whole blood (for IHI/Swiss TPH trial samples) and finally suspended in 50 μl or 30 μl of double-distilled sterile water, respectively. The template was kept frozen until needed. PCR amplification of template DNA and analysis of region II of *glurp*, central polymorphic region of *msp2 *(3D7 and FC27 allelic families), and block 2 of *msp1 *(K1, MAD20 and RO33 allelic families) was performed in accordance to the recently recommended genotyping protocol [23] with minor modifications, i.e. the primary PCR amplification for *glurp *and *msp2 *was duplexed whereas *msp1 *and all nested amplifications were uniplexed.

The primary duplexed and uniplexed PCR amplifications of were carried out in 75 μl whereas all nested reactions were uniplexed and done in 50 μl final reaction volumes. The primary duplexed (*glurp *and *msp2*) reactions contained 1x PCR buffer B (Firepol Solis^®^), 1.57 mM MgCl_2_, 120 μM of each dNTP, 0.4 units of Taq DNA polymerase (Firepol Solis ^®^), 30 μM of each oligonucleotide primer (Operon, Table 1) and 5 μl of template DNA.

**Table 1:** Name, sequence and amplicon sizes generated by *Plasmodium falciparum msp1, msp2 *and *glurp *PCR amplification primers

Gene	Amplification	Primer	Primer sequence
*msp1*	Primary	M1OR	5'cttaaatagtattctaattcaagtggatca 3'
		MIOF	5'ctagaagctttagaagatgcagtattg 3'
	Secondary	MKR	5'gcttgcatcagctggagggcttgcaccaga 3'
		MKF	5'aaatgaagaagaaattactacaaaaggtgc 3'
	Secondary	M1MR	5'atctgaaggatttgtacgtcttgaattacc 3'
		M1MF	5'aaatgaaggaacaagtggaacagctgttac 3'
	Secondary	M1RF	5'taaaggatggagcaaatactcaagttgttg 3'
		RO33R2	5'caagtaattttgaactctatgttttaaatcagcgta 3'
*msp2*	Primary	S2	5'-gaaggtaattaaaacattgtc 3'
		S3	5'-gagggatgttgctgctccacag 3'
	Secondary	S1TAIL FW	5'-gcttataatatgagtataaggagaa 3'
		M5-FC27-RV	6FAM 5'-gcattgccagaacttgaa 3'
		N5-3D7-RV	VIC 5'-ctgaagaggtactggtaga 3'
*glurp*	Primary	GF3	5'acatgcaagtgtgatcctgaa 3'
		GF4*	5'tgtaggtaccacgggttcttgtgg 3'
	Secondary	GNF	5'tgttcacactgaacaattagatttagatca 3'

Note: * used also for as reverse primer in the secondary amplification.

The nested *glurp *amplification final reaction volumes contained 1x buffer B, 1.5 mM MgCl_2_, 128 μM of each dNTP, 75 μM of each oligonucleotide primer, 0.4 units of Taq DNA polymerase (Firepol Solis ^®^) and 2 μl of primary PCR product as a template whereas nested *msp2 *reactions had 1x buffer B, 1.5 mM MgCl_2_, 200 μM of each dNTP, 300 μM of each fluorescent labeled family-specific oligonucleotide primers (Applied Biosystems) and 2 μl of primary PCR product as a template.

The uniplexed primary *msp1 *final reaction conditions were same as in *msp2*. The three nested *msp1 *family-specific amplification for Ro33-, K1- and MAD20-families each contained 1x PCR buffer B, 2 mM MgCl_2_, 200 μM of each dNTP, 0.4 units of Taq DNA polymerase, 25 μM of each oligonucleotide primer and 2 μl of primary *msp1 *amplification product as a template.

Thermo cycling was done using the MJ Thermal Controller PTC-100™ (MJ Research Inc. Watertown, USA). Temperature cycling parameters were: initial denaturation at 95°C for 5 minutes followed by 30 cycles of denaturation at 94°C for 1 minute, annealing at 58°C for 2 minutes (for both uniplexed and duplexed primary PCR amplification) or 59°C for 2 minutes (for *msp1 *and *glurp *nested PCR) and extension at 72°C for 2 minutes followed by 10 minutes of final extension at 72°C. Nested PCR for *msp2*: initial denaturation was 94°C for 2 minutes followed by 30 cycles of denaturation at 94°C for 30 seconds, annealing at 50°C for 45 seconds and extension at 70°C for 2 minutes. The last extension cycle was prolonged for 10 minutes.

The primary and nested PCR amplification of the *msp1 *and *glurp *loci were carried out at IHI laboratory but all gel electrophoresis and digital image analysis were done at the Swiss TPH laboratory. For the *msp2 *locus, all primary amplification reactions were done at IHI laboratories whereas all nested PCR amplification and subsequent analysis were done in Basel and the conditions were as described elsewhere [19].

### Allele detection and estimation of molecular weight

The nested *glurp *and *msp1 *PCR products were separated directly on 3% or 3.5% ethidium bromide-stained agarose gels, respectively, visualized under UV illumination and photographed. Gel photographs were visually examined to identify successfully amplified fragment. All *glurp *and *msp1 *positive samples were separated again using 1.5% or 2% agarose gels, respectively, and directly analysed by a digitalized gel documentation system where band sizing and molecular weight were calculated by the AlphaEase^®^FC software version 6.0.0 Alpha Innotech. The fragments were then grouped into "bins" differing by 25 bp for *msp1 *and 50 bp for *glurp*. All fragments falling within the limits of the bin were considered to belong to the same genotype. Bins were determined manually and allelic frequencies were calculated by Stata v 9.0 (Stata Corporation Inc, Texas USA and Stat View version 5.0.1).

The nested *msp2 *amplification products were separated in 1.5% agarose gel in order to identify positive samples, which were subsequently prepared for capillary electrophoresis analysis. Briefly, PCR products were diluted with distilled water to the ratios 1:10, 1:20 and 1:30 according to the intensity of the bands in the nested PCR product. This was followed by the addition 10 ul of a 1:40 fluorescent size standard ROX-500 (Applied Biosystems) consisting of 16 fragments ranging for 50 -500 bp in length, dried and shipped to the Medical Research Center Genomic Facility-London, England) for capillary electrophoresis. At the genomic facility, samples were analyzed by automated 3730xls DNA analyzer and a GeneMapper^® ^software analyzer version 3.2. (Applied Biosystems LTD) was used to determine the size of PCR fragments, as previously described [19]. Analysis of *msp2 *raw data, allele calling according to manually selected 3 bp bins and calculation of a cut off and allelic frequencies was performed by dedicated software designed at the Swiss TPH.

#### Allelic richness and diversity and mean multiplicity of infection (MOI)

As a measure for genetic diversity, the expected heterozygosity (H_E_) was calculated by use of the formula H_E _= [n/(n-1)] [(1-∑P_i_^2^)], where n = sample size, Pi = allele frequency. The mean multiplicity of infection (MOI) was calculated as the quotient of the total number of *P. falciparum *genotypes for each marker in a particular country and the number of positive PCR samples.

## Results

### Study profile

Out of 780 baseline (Day 0) DNA samples, 599 (76.8%) were successfully amplified for *msp1*, 679 (87%) for *msp2 *and 575 (73.7%) for *glurp *loci (Table 2).

**Table 2:** DNA amplification success rate for each marker gene in baseline samples in 5 countries

Country	Study year	n	PCR-positive samples					
			***msp1 *(%)**				***msp2 *(%)**	***glurp *(%)**
			**K1**	**MAD20**	**RO33**	**Total**		

B*. Faso	2000	180	71	33	37	95 (53)	154 (86)	110 (61)
Malawi	2000	180	100	53	68	146(81)	145 (81)	135 (75)
São Tomé	2000	180	100	65	65	171 (95)	164 (91)	145 (81)
Tanzania	1996	60	48	30	10	50 (83)	56 (93)	45 (75)
Uganda	2000	180	100	41	27	137 (76)	160 (89)	140 (78)

Total		780				599(76.8)	679 (87)	575(73.7)

Note: * B = Burkina, S = Study

### Mean multiplicity of infection (MOI)

Table 3 shows mean MOIs calculated using data from each of the three marker genes for each of the five study countries. The mean MOI for *msp2 *loci was the highest (2.24), followed by that for *msp1 *(1.48) and then for *glurp *(1.4). The mean MOI values calculated for *msp1 *and *glurp *were similar but substantially different from those of *msp2*.

**Table 3:** Mean Multiplicity of infection recorded using *msp1, msp2 *and *glurp *loci in the 5 study countries

	Malawi	B. Faso	Sao Tome	Tanzania	Uganda	Average
*msp1*	1.03	1.40	1.30	2.50	1.18	1.48
*msp2*	1.52	3.03	2.00	3.48	1.17	2.24
*glurp*	1.01	1.86	1.01	1.84	1.29	1.40

Study participant were children 0 to 5 years old at all sites.

Note: B = Burkina

### Genetic diversity and allelic frequency

**Figure 1:**
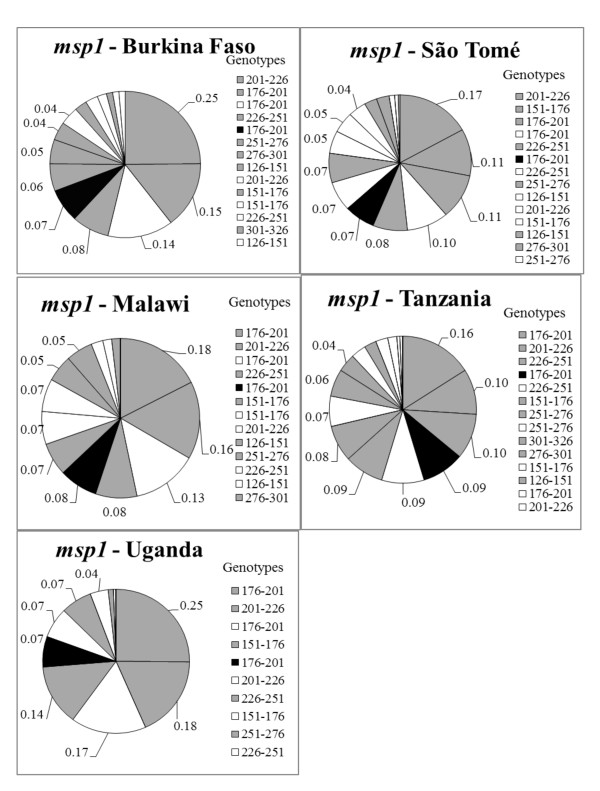
***msp1 *allelic frequency (%) by country**. Most of the genotypes occurred below 10%. Four K1-family genotypes and two MAD20-family genotypes occurred above 10%. Note; only genotypes with frequency ≥ 4% (0.04) are labeled.

The number of genotypes observed at each marker by country is shown in Table 4. Seventeen (17) different *msp1 *genotypes were observed, representing K1 (8 genotypes), MAD20 (8 genotypes) and RO33 (1 genotype) allelic families. The *msp 1 *fragment sizes ranged from 135 bp - 305 bp. Figure 1 shows the frequencies of *msp1 *genotypes by country. The majority of these genotypes occurred at a frequency below 10%. However, four genotypes from the K1 allelic family (176 bp-201 bp, 201 bp-2262 bp, 226-251 bp, 151-176 bp), two from the MAD20 family (176 bp-201 bp and 201 bp-226 bp) occurred above 10%.

**Table 4:** *msp1, msp2 *and *glurp *alleles observed in the 5 study countries

Country	Number of genotypes per allelic-family/locus							
	***msp1***				***msp2***			***glurp***
	**K1 (%)**	**Mad20 (%)**	**RO33 (%)**	**Total**	**3D7 (%)**	**FC27 (%)**	**Total**	

B**. Faso	8(53.3)	6(40)	1(6.7)	15	71 (73.2)	26 (26.8)	97	14
São Tomé	8(50)	7(43.7)	1(6.3)	16	60 (80)	15 (20)	75	14
Malawi	7(53.8)	5(38.5)	1(7.7)	13	53 (76.8)	16 (23.2)	69	13
Uganda	5 (50)	4 (40)	1 (10)	10	38 (77.6)	11 (22.4)	49	10
Tanzania	8(50)	7(43.8)	1(6.20)	16	57 (78.1)	16 (21.9)	73	13

Total*	8	8	1	**17**	83	33	**116**	**14**

Note: * Number of observed allele not sum of alleles, ** B = Burkina

The R033 family is considered to be monomorphic with a predicted amplified fragment size of 215 bp. However, the gel analysis software estimated the fragment amplified from most samples to be between 180 bp - 210 bp. When a set of 6 samples representative of these two sizes were sequenced, they were all found to be 215 bp long.

**Figure 2:**
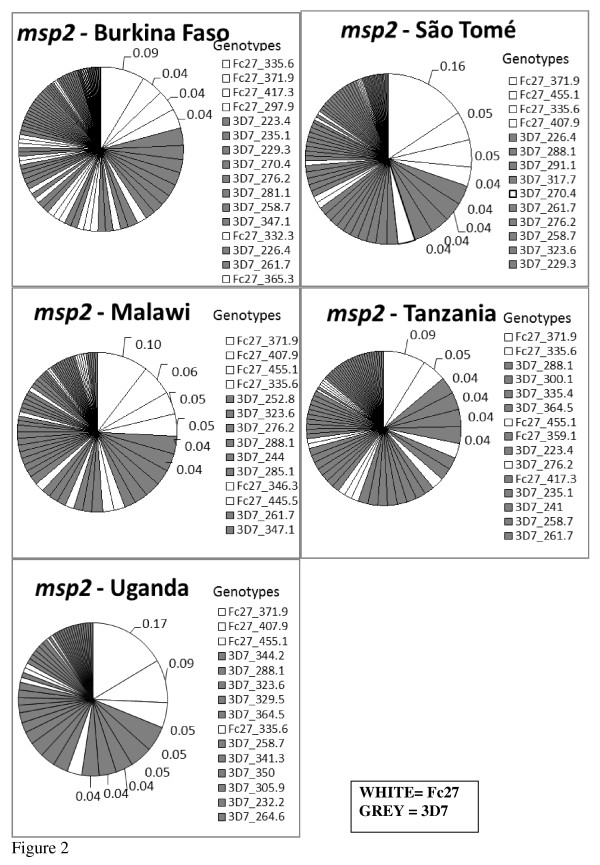
***msp2 *allelic diversity and frequency (%) by country**. Only three genotypes, all from FC27 family occurred at of above 5%. Note; only genotypes with frequency ≥ 4% (0.04) are labeled.

The *msp2 *diversity and genotype frequencies for each of the countries are shown in Figure 2. A total of 116 different *msp2 *genotypes (size range from 205 to 518 bp) were recorded at the 5 study sites, of which, 83/116 (71.6%) and 33/116 (28.4%) belonged to the 3D7 and FC27 allelic families, respectively (Table 4). The frequencies of individual *msp2 *genotypes were low with 96.5% occurring at a frequency ≤ 5%. Only four (3.5%) genotypes, all belonging to the FC27 allelic family were found at frequencies at or above 5%.

**Figure 3:**
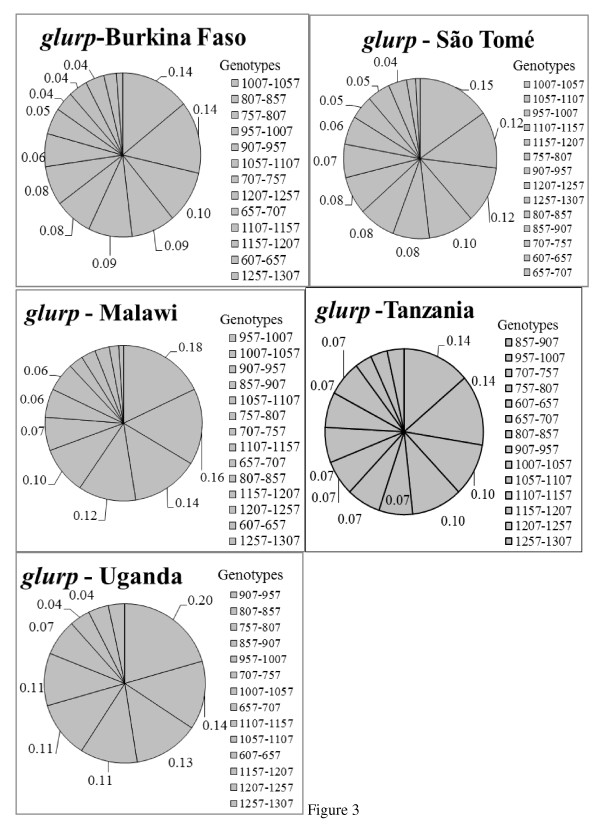
***glurp *allelic diversity and frequency (%) by country**. Only genotypes with frequency ≥ 4% (0.04) are labeled.

Fourteen (14) different *glurp *genotypes were detected and their diversity across the countries is shown in Figure 3. The allelic variants ranged between 650 bp and 1,250 bp, and the majority (77.4%) occurred at frequencies < 10%. Two genotypes, bin sizes 907 bp-957 bp and 957 bp-1,007 bp, had the highest frequencies, 20% in Uganda and 18% in Malawi, respectively.

Expected heterozygosity (H_E_) was calculated in order to assess the discriminatory power of the three individual markers. H_E _estimates the fraction of all parasites that would be heterozygous for any of the three loci. Table 5 shows generally very high H_E _values across the five sites studies.

**Table 5:** Genetic diversity of *msp1, msp2 *and *glurp *measured as expected heterozygosity (H_E_)

**H**_**E **_**= [n/(n-1)] [(1-∑P**_**i**_^**2**^**)]**					
	Malawi	Burkina Faso	São Tomé	Tanzania	Uganda
*msp2*	0.97	0.98	0.96	0.99	0.95
*msp1*	0.79	0.78	0.83	0.84	0.68
*glurp*	0.89	0.91	0.91	0.92	0.88

## Discussion

Polymorphic regions from of the *P. falciparum msp1, msp2 *and *glurp *loci have been selected as the recommended markers for parasite genotyping in anti-malarial drug trials and efficacy studies [23]. However, the parasites' genetic profile has not been systematically documented in many malaria endemic countries. A large number of archived *P. falciparum *positive pre-treatment infections were genotyped in order to compare the diversity and allelic frequencies for these three markers among five geographical areas with different transmission intensities across sub-Saharan Africa; namely Malawi, Tanzania, Uganda, Burkina Faso and São Tomé. These blood samples were collected in the region during two clinical trials (WHO/TDR and IHI/Swiss TPH) of artemisinin-based combination therapy that were conducted between 1996 and 2000. The findings from these studies have been published elsewhere [22,13]. The aim of this study was to determine whether the genetic diversity of the markers or their suitability for PCR-correction of drug efficacy trials in endemic countries, varied between countries. In addition, since the PCR-corrected treatment failures in both of the above trials were >10%, the study also aimed at validating these corrections by assessing the Day 0 genotypic profile as recommended [23].

Although Isocode stix were stored at room temperature over nine years, the majority (53 - 95%) of the DNA samples could be amplified; with some unexplained variation unrelated to amplified fragment size across the geographical sites. This molecular genotyping study shows that on average the majority of the patients were infected with more than one parasite genotype on the day of admission. The mean MOI values were heterogenous across the different sites, being lowest in Uganda, and highest in Burkina-Faso and Tanzania. Mean MOI was highest for *msp2 *(2.24), followed by *msp1 *(1.48) and was lowest for *glurp *(1.40) and the allelic diversity followed a similar trend recording 116, 17 and 14 alleles, respectively. The allelic variants were spatially distributed across the five Sub-Saharan African countries. The differences in allele diversity between *msp2 *on the one hand and *msp1 *and *glurp *on the other are clearly attributable to the method used for DNA fragment sizing. Indeed capillary electrophoresis used for *msp2 *has a much higher power of resolution than gel elecrophoresis and digitalized fragment sizing used for *msp1 *and *glurp *[24]. However, since the genotyping methods used were the same for all geographic sites, it is possible to compare diversity of a given marker between countries. Tanzania recorded the highest genetic diversity, while Uganda recorded the lowest diversity in all the three markers.

In the WHO/TDR trials [15], the crude post day-14 parasitological recurrence rate for all treatment groups was 22% and was 89.7% for chloroquine alone and 13.6% for artemether-lumefantrine for the IHI-Swiss TPH trial [21]. According to the recently adopted recommendations for malaria genotyping [23], PCR-corrected failure rates exceeding 10% would require determining MOI and allelic frequencies in order to confirm the validity of the PCR-correction. Thus observation of high MOI, high allele diversity and low allele frequencies for all markers (especially *msp2 *whose fragments were sized by the most accurate method) and study sites are strongly indicative of the high discriminatory power of this three-marker genotyping strategy. These findings further validate the PCR-adjusted outcomes recorded previously [15,21].

High genetic diversity and low allelic frequencies have been reported previously from other sites that differ substantially in transmission intensity: Gabon [7], Uganda [10], Senegal [25], Burkina Faso [26] and Honduras [27]. Nonetheless, three allelic variants of *msp1 *(151-176 bp, 176-201 bp and 201-226 bp), four of *msp2 *(wos12, wos3, K1-long and FC27-455) and five of *glurp *(857-907 bp, 907-957 bp, 957-1007 bp, 1007-1057 and 1057-1107 were recorded at frequencies exceeding 5% in some of the study sites. Such frequently occurring genotypes might lead to misclassification of recurrent parasites in clinical trials and drug efficacy studies. However, from the current data even the most abundant genotype of *glurp *with a recorded allelic frequency of 20%, the probability of acquiring a new independent infection of the same genotype by chance equals the square of the allelic frequency. This probability of 4% for a reinfection with the same genotype is not very high for a single genotyping marker; it would be even much lower when all three markers were genotyped.

It should be noted that the blood samples analysed were collected more than nine years ago, hence the genetic profiles described might no longer accurately represent the current situation. Nonetheless, this study provides important genetic background data in these areas. The strength of the present study hinges on four major factors: (i) the large number of baseline samples that, (ii) were collected from five countries with different levels of transmission intensity, (iii) use of automated DNA sizing methods to remove investigator bias and error in assigning molecular weights of the PCR products, and (iv) comparative analyses across countries could be conducted, because the data was obtained using the same amplification protocol.

One of the factors that directly impinge on the utility of genotyping protocols in drug efficacy studies is that of bin size selection. For this study, as for all previous studies, the bin sizes for *msp1 *or *glurp *fragments have been set rather arbitrarily. The lengths of the repeat units, whose number varies between the different allelic variants, was taken into account to set bin width of 25 bp and 50 bp for *msp1 *and *glurp*, respectively. In contrast, *msp2 *fragments were sized by capillary electrophoresis with 3 bp bin width that quite obviously represents the smallest size difference possible in a coding region. In previous studies, different bin widths were used, for example, [28] used a conservative bin width of 40 bp for *msp1 *and *msp2*, whilst [10] used bin sizes of 10 bp for *msp1 *and *msp2 *and 20 bp for *glurp*. In addition to these variations in bin width, the different fragment sizing methods employed by different researchers make it difficult to compare data for a particular marker between studies.

Another drawback of this genotyping protocol is the variability in the electrophoretic migration of a given DNA fragment between gels, and indeed between different regions of the same gel. This is clearly illustrated in our observations of variable RO33 fragment sizes by digital gel documentation, despite the fact that these fragments were confirmed to be of the same size (215 bp) by sequencing. It is most likely that such spurious variability will also occur for variants of the other *msp1 *allelic families, and maybe even to a greater extent in the much larger *glurp *fragments. Thus, the quality and value of the data obtained from binning of alleles across different gels depends largely on gel quality (e.g. "smiling effect" or unequal loading of gel slot) and on the accuracy of image digitization. Electrophoretic variability is a potential problem when one wishes to establish the frequencies of each allelic variant. When sizing is done across different gels, binning through an image analyzer might be more prone to error than doing it by eye, especially when samples are run side by side. This error observed when comparing fragments from different gels, is highly unlikely to affect the validity of the genotype pattern comparisons of baseline and recurrent infections, on which PCR correction is based, because the amplified products from these samples are usually migrated in the gels side-by-side and, therefore, with a much reduced chances of variability. For such comparisons, detection of size differences can either be done visually or through an image analysis programme. Admittedly, visual analysis also poses some degree of subjectivity: at what level of difference in migration does one say that two bands are different, especially when they differ in quantity and consequently intensity and thickness in the gel.

Ultimately, the ability to distinguish between two allelic variants depends directly on the resolution of the method used to analyse the amplified fragments. At present capillary electrophoresis offers the best solution, because it provides accurate and reproducible estimates of DNA fragment lengths with a resolution power down to a few base pairs difference. It is highly likely that the *msp1 *and *glurp *fragments amplified in the course of this study in fact encompassed a larger repertoire of distinct allelic variants than those resolved by simple agarose gel electrophoresis. By the time this study was done, only *msp2 *capillary electrophoresis protocol was described [19]. Now protocols for *msp1 *[29,30] and *glurp *[24] have been developed and all three markers can be analyzed and allele diversity/frequency compared more accurately.

## Conclusion

The *P. falciparum msp1, msp2 *and *glurp *markers used for PCR-correction of treatment outcomes in the context of drug clinical trials or efficacy studies in endemic areas appear to be highly polymorphic and to have low allelic frequencies across sub-Saharan countries with varying transmission intensities. These observations reinforce the value of these genotyping markers in classifying recurrent post-treatment *P. falciparum *episodes as recrudescence or new-infections. However, these findings strongly suggest that standardized protocols should include optimal methods for fragment size estimation and bin width determinations. This will further enable and simplify data comparison between sites/studies. With the expanding access to ACT and current changes in malaria epidemiology, the *P. falciparum msp1*, *msp2 *and *glurp *allele frequency/genetic diversity should be monitored regularly to ensure the reliability of the PCR-adjusted treatment outcome.

## Statement of conflict of interest

The authors do not have any commercial or other association that may pose conflicts of interest concerning the work reported in this paper.

## Authors' contributions

Study conceptualization and design was done by KM, HPB and PO. KM and IF did study monitoring and supervision in Tanzania and Switzerland, respectively. IF and GS provided technical advisory support in genotyping and data interpretation. FM, DS, SS and KM carried out the laboratory work. FM and GN performed statistical analysis. FM, GN, and KM composed the primary version of the manuscript and all other authors contributed modifications. All authors read and approved the final manuscript.
